# Bile acids as a key target: traditional Chinese medicine for precision management of insulin resistance in type 2 diabetes mellitus through the gut microbiota-bile acids axis

**DOI:** 10.3389/fendo.2024.1481270

**Published:** 2024-12-10

**Authors:** Yu Wang, Jing Yu, Binqin Chen, Wenqi Jin, Meili Wang, Xuenan Chen, Mengqiong Jian, Liwei Sun, Chunli Piao

**Affiliations:** ^1^ College of Traditional Chinese Medicine, Changchun University of Chinese Medicine, Changchun, Jilin, China; ^2^ Department of Endocrinology, the Affiliated Hospital of Changchun University of Chinese Medicine, Changchun, Jilin, China; ^3^ Applicants with Equivalent Academic Qualifications for Master Degree, Guangzhou University of Chinese Medicine, Guangzhou, Guangdong, China; ^4^ Shenzhen Hospital (Futian), Guangzhou University of Chinese Medicine, Shenzhen, China; ^5^ Research Center of Traditional Chinese Medicine, the Affiliated Hospital of Changchun University of Chinese Medicine, Changchun, Jilin, China; ^6^ Northeast Asian Institute of Traditional Chinese Medicine, Changchun University of Chinese Medicine, Changchun, Jilin, China

**Keywords:** bile acids, the gut-liver axis, insulin resistance, type 2 diabetes mellitus, traditional Chinese medicine

## Abstract

Type 2 diabetes mellitus (T2DM) is a chronic metabolic disease caused by insulin resistance (IR) and insufficient insulin secretion. Its characteristic pathophysiological processes involve the interaction of multiple mechanisms. In recent years, globally, the prevalence of T2DM has shown a sharp rise due to profound changes in socio-economic structure, the persistent influence of environmental factors, and the complex role of genetic background. It is worth noting that most T2DM patients show significant IR, which further exacerbates the difficulty of disease progression and prevention. In the process of extensively exploring the pathogenesis of T2DM, the dynamic equilibrium of gut microbes and its diverse metabolic activities have increasingly emphasized its central role in the pathophysiological process of T2DM. Bile acids (BAs) metabolism, as a crucial link between gut microbes and the development of T2DM, not only precisely regulates lipid absorption and metabolism but also profoundly influences glucose homeostasis and energy balance through intricate signaling pathways, thus playing a pivotal role in IR progression in T2DM. This review aims to delve into the specific mechanism through which BAs contribute to the development of IR in T2DM, especially emphasizing how gut microbes mediate the metabolic transformation of BAs based on current traditional Chinese medicine research. Ultimately, it seeks to offer new insights into the prevention and treatment of T2DM. Diet, genetics, and the environment intricately sculpt the gut microbiota and BAs metabolism, influencing T2DM-IR. The research has illuminated the significant impact of single herbal medicine, TCM formulae, and external therapeutic methods such as electroacupuncture on the BAs pool through perturbations in gut microbiota structure. This interaction affects glucose and lipid metabolism as well as insulin sensitivity. Additionally, multiple pathways including BA-FXR-SHP, BA-FXR-FGFR15/19, BA-FXR-NLRP3, BA-TGR5-GLP-1, BAs-TGR5/FXR signaling pathways have been identified through which the BAs pool significantly alter blood glucose levels and improve IR. These findings offer novel approaches for enhancing IR and managing metabolic disorders among patients with T2DM.

## 1 Introduction

The intricate and interdependent relationship between the gut-liver axis and the host has emerged as a prominent area of research in the biomedical field in recent years (1–4). This symbiotic balance exerts a profound influence on human health maintenance and disease onset and progression. Notably, the gut microbiota-bile acids (BAs) axis plays a pivotal role in regulating host immunity, glucose metabolism, and lipid homeostasis, garnering increasing recognition from academia (5–7). The gut microbiota plays a pivotal role in this axis, exerting profound influence on the biological activity of BAs through their transformation and modification (8). Subsequently, BAs function as signaling molecules via nuclear and membrane binding receptors to execute diverse metabolic functions (9). Therefore, the gut microbiota-BAs axis is considered an “endocrine organ” capable of influencing the host’s physiological state (10, 11). Recent studies have shown that the gut microbiota-BAs axis also plays a key role in insulin resistance (IR) in type 2 diabetes mellitus (T2DM) (12, 13). The available evidence indicates a significant global increase in the prevalence of diabetes, with IR being prevalent among the majority of diabetic patients (14).

T2DM manifests a diverse array of clinical presentations, primarily due to alterations in pancreatic islet function, IR, metabolic derangements, and other related changes (15, 16). It encompasses a multifaceted etiology, including not only immune dysfunction and genetic predisposition but also crucial lifestyle factors and obesity, which play pivotal roles in its development, consequently resulting in disturbances in carbohydrate, lipid, fluid-electrolyte balance, and protein metabolism (17–20). The prevalence of T2DM is on the rise, indicating a significant inclination towards younger age groups (21). The estimated global prevalence of diabetes among individuals aged 20-79 years in 2021 stood at a concerning rate of 10.5% (22). T2DM, as a chronic condition, exerts detrimental effects on multiple organs, including blood vessels, eyes, kidneys, and even the feet, in the absence of effective management (23). Its uncontrolled state can lead to substantial disability and mortality rates, thereby imposing a heavy burden on healthcare expenditure, with projections indicating that by 2045, the economic cost associated with T2DM will escalate to an astonishing $1,054 billion (22).

Prediabetes, a precarious condition that signifies a heightened risk for the progression to T2DM and serves as a reservoir for the burgeoning diabetic population, has attained a staggering prevalence rate of 50.1% (24). This preclinical condition is fundamentally characterized by IR (25). IR refers to the diminished response of target tissues to the action of insulin, resulting in different levels of impaired glucose and lipid metabolism accompanied by elevated levels of inflammatory mediators (26). Currently, despite the management of prediabetes predominantly revolving around lifestyle modifications, there exists considerable variability in individual responses and adherence. Against this backdrop, Traditional Chinese Medicine (TCM) has gradually emerged as a promising complementary and alternative approach in the prevention and treatment of prediabetes, leveraging its distinct theoretical framework and extensive clinical experience (27–29). By employing a multimodal approach that includes herbal formulas, acupuncture, tuina, and other therapeutic techniques, TCM aims to harmonize yin and yang, unblock meridians, strengthen spleen and kidney functions, thereby enhancing constitutional health and bolstering the innate regulatory mechanisms, ultimately preventing or delaying the onset and progression of T2DM (30, 31).

Therefore, the exploration of novel therapeutic strategies targeting the gut microbiota-BAs axis, particularly those anchored in TCM, holds immense promise for the precision management of IR in T2DM. By delving deeply into the intricate interplay between the gut microbiota, BAs, and IR, our research thus aims to elucidate the mechanisms underlying the efficacy of TCM in modulating this axis and thereby mitigating the progression of T2DM. Understanding the role of the gut microbiota-BAs axis in the pathogenesis of T2DM and the modulation of IR through interventions based on TCM not only advances our knowledge of the complex etiology of the disease but also paves the way for the development of innovative, personalized treatment strategies.

## 2 BAs

The synthesis and metabolism of BAs constitute a sophisticated and finely tuned process, necessitating the concerted efforts of the liver, intestine, and gut microbiota. BAs can be categorized structurally into free and conjugated forms, while their origins are delineated as primary and secondary BAs. To deepen our exploration of the gut microbiota-BAs axis in T2DM and its modulation by TCM, it becomes essential to elucidate the fundamental mechanisms underlying BAs synthesis and metabolism. This intricate interplay among the liver, gut, and gut microbiota lays the groundwork for comprehending how alterations in BAs composition can impact IR and subsequently drive the progression of T2DM.

### 2.1 Production and metabolism of BAs

BAs synthesis and metabolism is a complex and finely regulated process involving a synergistic interaction of the liver, gut, and gut microbes (32). BAs are synthesized by hepatocytes utilizing cholesterol as a substrate, starting from two distinct biosynthetic pathways, namely the classical pathway initiated by the rate-limiting enzyme cholesterol 7α-hydroxylase (CYP7A1), and the alternative pathway initiated by sterol 27-hydroxylase (33, 34). In the synthesis of total BAs, the classic pathway predominates, accounting for over 70% of production (35) and contributing approximately 5 g of BAs daily (36). These BAs subsequently conjugate with glycine and taurine to form salts, with a conjugation ratio of approximately 3:1 for glycine to taurine (37). Bile salt export pump (BSEP) serves as the principal BAs efflux transporter, cooperating with multi-drug resistance protein (MDR3) and protein contributing to membrane lipid composition (FIC1) to shuttle BAs from hepatocytes into bile canaliculi (38).

**Figure 1:**
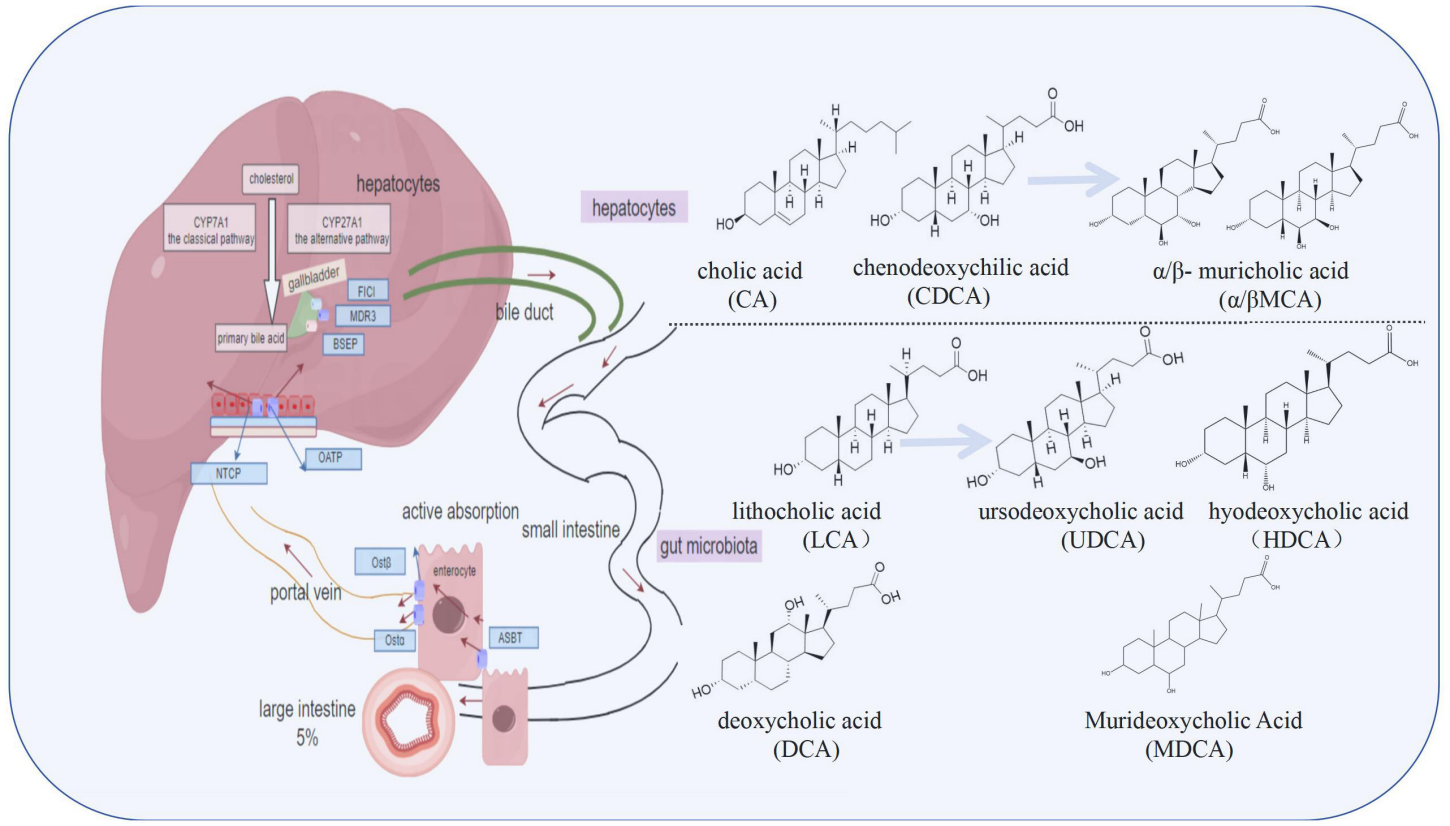
The synthesis and metabolic cycle of bile acids (BAs) involve the liver, intestine, and gut microbiota. BAs are synthesized in hepatocytes via the classic pathway and the alternative pathway, and are primarily conjugated with glycine and taurine before being transported to the bile duct by transporters such as bile salt export pump (BSEP). They are stored in the gallbladder and further transformed by gut microbiota into secondary bile acids like lithocholic acid (LCA), deoxycholic acid (DCA), and ursodeoxycholic acid (UDCA), increasing their diversity and complexity. Most bile acids are reabsorbed through passive diffusion or active transport via apical sodium-dependent bile acid transporter (ASBT) in the ileum, entering the portal circulation through the heterodimeric organic solute transporter (OSTα/β), and then transported back to hepatocytes by Sodium/taurocholic acid cotransport polypeptide (NTCP) and organic anion transporting polypeptide (OATP), completing the enterohepatic circulation. Unabsorbed bile acids are excreted in feces and urine.

Upon secretion into the gallbladder via the bile duct, BAs are stored awaiting the digestive stimulus of food intake. When food enters the small intestine, the gallbladder contracts, releasing BAs into the duodenum to facilitate the emulsification and absorption (39). Within the gut microbiota environment, primary BAs undergo debinding, dehydroxylation, and epimerization processes to generate secondary BAs such as LCA, deoxycholic acid (DCA), and UDCA (40, 41). Notably, in the realm of secondary BAs, LCA can undergo conversion to UDCA via 7a-hydroxylation, hyodeoxycholic acid through 6a-hydroxylation or murideoxycholic acid by means of 6β-hydroxylation, thereby augmenting the diversity and intricacy of BAs composition (41–43). A portion of BAs is reabsorbed via passive diffusion mechanisms in the small and large intestines, while another fraction undergoes active absorption at the apical membrane of the ileum through the apical sodium-dependent bile acid transporter (ASBT), subsequently entering the portal circulation via the heterodimeric organic solute transporter (OSTα/β) complex (44–46). Approximately 95% of BAs are efficiently reabsorbed in the ileum and transported back to hepatocytes by Sodium/taurocholic acid cotransport polypeptide (NTCP) and organic anion transporting polypeptide (OATP), completing their enterohepatic circulation and metabolism (3, 47). Unabsorbed BAs proceed to the large intestine, with the remainder excreted in feces and urine (48). Under normal physiological conditions, the enterohepatic circulation occurs 8 to 10 times daily (49). In conclusion, the synthesis and metabolism of BAs constitute a highly intricate and precisely regulated system that highlights the harmonious collaboration between the liver, gut, and gut microbiota, ensuring efficient lipid digestion, absorption, and maintenance of metabolic homeostasis. **Figure 1** illustrates the process of BAs metabolism.

### 2.2 BAs and metabolic disorders

BAs, serving as vital signaling molecules between the liver and intestine, play pivotal roles in regulating energy metabolism and maintaining intestinal microecology homeostasis (50). Synthesized in the liver and secreted into the intestine, BAs exert profound influences on the intestinal microbial community structure (51). IR, a hallmark of T2DM, promotes fat accumulation in the liver through multiple pathways, leading to liver damage and subsequently affecting BAs synthesis and secretion (52). BAs are potent antibacterial compounds that play an important role in shaping the ecology of gut microbes (53). The diversity of bacteria in the intestines can tolerate the antibacterial activity of bile salts through a variety of physiological adjustments, and the resistance to bile salts allows certain bile-resistant pathogens to colonize the liver and biliary tract (54). When BAs metabolism is disrupted, resulting in alterations in the gut microbiota composition, increased intestinal permeability, and the entry of more harmful bacteria and endotoxins into the portal vein, further exacerbating liver damage (55). In addition, activation of bile acid receptors triggers mediates the secretion of glucagon-like peptide-1 (GLP-1) by enteroendocrine cells (56). GLP-1 reduces gluconeogenesis, increases energy expenditure, stimulates pancreatic insulin secretion, and attenuates inflammatory responses in immune cells (57, 58). In the context of IR, abnormalities in BAs metabolism may exacerbate liver damage and the risk of metabolic diseases by affecting the intestinal microecology.

BAs can be classified into 12α-hydroxylated and non-12α-hydroxylated forms based on their chemical structure, with distinct implications for metabolic health. The main constituents of 12α-OH BAs are cholic acid (CA) and chenodeoxycholic acid (CDCA), and their conjugates with taurine or glycine through amide bonds, while non-12α-hydroxylated BAs include CDCA, Lithocholic acid (LCA), and ursodeoxycholic acid (UDCA) (59, 60). Research has demonstrated that an elevated ratio of 12α-OH BAs to non-12α-OH BAs in serum, which is associated with T2DM, obesity, and IR, may be attributed to an underlying mechanism whereby abnormally elevated glucose and insulin levels induce histone acetylation on the chromatin of CYP7A1, thereby stimulating CYP7A1 synthesis and subsequently leading to an upregulation in the production of 12α-hydroxylated BAs (60–62).

Further observation showed that significant differences were observed in the composition of BAs among the liver, gallbladder, intestine, feces, and serum. There exist notable distinctions between mice and humans, particularly in terms of their BAs composition. Mice exhibit significantly elevated levels of hydrophilic BAs compared to humans (49), which can be primarily attributed to the conversion pathway of CDCA to α/β- muricholic acid (MCA) in mice, leading to a higher proportion of hydrophilic BAs (63). It is worth noting that, while Hyocholic acid (HCA) and its derivatives are present in very low amounts in human blood, they constitute approximately 76% of the total BAs pool in pigs, which have garnered attention due to their remarkable resistance to T2DM (56). These differences not only reflect the species-specific nature of BAs metabolism but also offer new perspectives for studying and understanding the role of BAs in physiology and disease.

BAs is a kind of amphiphilic steroid acid, whose production and diversity are influenced by both host and microbial metabolism (3, 39). Hydrophilic BAs exhibit a certain degree of hepatoprotective effect, whereas hydrophobic BAs possess cytotoxicity, leading to the accumulation of BAs and subsequent damage, necrosis, and apoptosis in liver cells. This disruption can significantly influence gut microbial homeostasis, subsequently affecting overall organismal health. Hydrophobic BAs are considered potential biomarkers for early detection of metabolic disorders. Obese mice demonstrate significantly elevated levels of hydrophobic BAs in both ileal contents and feces compared to normal mice (64). It is noteworthy that hydrophobic BAs possess the ability to inhibit intestinal cholesterol absorption in a high-fat diet context. As the concentration of hydrophobic BAs increases, it consequently imposes a greater metabolic burden on the liver, thereby further escalating the risk of developing metabolic disorders (52). Interestingly, mice with the cytochrome P450 family 2 subfamily c polypeptide 70 knockout display a more hydrophobic composition of BAs pool resembling that of humans (65). Therefore, monitoring and analyzing hydrophobic BAs play a crucial role in the prevention and treatment of metabolic disorders. These findings not only enhance our comprehension of the disparities in BAs metabolism between rodents and humans but also have significant implications for utilizing mouse models to investigate BAs-related diseases in humans.

## 3 T2DM-IR and structural changes of gut microbiota

In regulating host metabolism, the gut microbiota plays a pivotal role, particularly as it exerts its influence through intricate and finely tuned interactions that affect multiple facets, including insulin signaling, glucose and lipid metabolism, and protein metabolism, all of which are crucial in the onset and progression of IR (66). Homeostatic model assessment of insulin resistance (HOMA-IR) is an indicator of IR, with its levels directly influenced by the composition of the gut microbiota (67). Specifically, three bacterial taxa, namely Cyanobacteria, Acidobacteria, and Gemmatimonadetes, exhibited a negative correlation with HOMA-IR, suggesting their potential to ameliorate IR (68). Further research also pointed out that effective treatment interventions can reinstate the abundance of microbiota, thereby ameliorating associated symptoms (69–71). It is noteworthy that the improvement of insulin sensitivity by metformin, a widely used hypoglycemic drug, is closely related to the regulation of gut microbiota, demonstrating the complexity of drug-microbial interactions (72). Sun et al. found that metformin can effectively prevent and treat impaired glucose tolerance and IR by inhibiting intestinal the Farnesoid X receptor (FXR) signal through the gut microbiome in a non-AMP-activated protein kinase (AMPK)-dependent manner (73). This finding not only deepens our understanding of the mechanism of action of metformin, but also highlights the importance of gut microbiota in the efficacy of the drug. Furthermore, TCM, with its unique theoretical framework and therapeutic advantages, has demonstrated considerable potential in the treatment of chronic metabolic diseases associated with IR (74, 75). In recent years, the gut microbiota has emerged as a novel bridge linking TCM therapies to disease improvement, becoming a frontier and hot research area in exploring the mechanisms of action of Chinese medicinal herbs (76).

TCM boasts a rich history in the management of T2DM. As research progresses, an increasing body of evidence demonstrates that TCM can effectively ameliorate IR through the regulation of gut microbiota (77–79). The compound salvianolic acid A, derived from Salvia miltiorrhiza and recognized as an effective therapeutic agent for T2DM, not only demonstrates significant effects on modulating the gut microbiota in T2DM-IR rats, but also notably enhances the richness and diversity of this microbiota when administered at a high dose, thereby promoting homeostasis of the core gut microbiota (80). Lycium barbarum polysaccharide, similar to Salvia miltiorrhiza, not only alleviates hyperglycemia, hyperlipidemia, and IR in diabetic mice but also highlights the crucial role of gut microbiota in regulating IR, as evidenced by the negative correlation between the presence of *Cetobacterium*, *Millionella*, *Clostridium*, *Streptococcus*, and *Ruminococcaceae* and HOMA-IR (81). The application of licorice extract demonstrated positive effects, effectively ameliorating various symptoms in diabetic mice in a dose-dependent manner and exerting its effects through gut microbiota remodeling (82). The investigation of Puerariae Lobatae Radix in db/db mice demonstrates its potential to effectively ameliorate the inflammatory damage inflicted upon islet cells and mitigate IR, potentially attributed to its ability to enhance the abundance of specific gut microbiota and regulate the expression of metabolite-associated proteins within the gut microbiota (83). Simultaneously, emerging therapeutic approaches such as targeted probiotic supplementation and fecal transplantation demonstrate potential in modulating the gut microbiota composition to prevent and treat IR in individuals with T2DM (84, 85). In the management of T2DM-IR patients, interventions such as administration of multiple prebiotics, probiotic strains or fecal microbiota transplantation exhibit a substantial amelioration in HOMA-IR indices and exert a favorable impact on glycemic control (86–88). The findings not only validate the application of TCM in treating T2DM, but also underscore its significant potential in ameliorating IR (89, 90).

## 4 T2DM-IR and gut microbiota-mediated BAs metabolism

Current studies have shown that gut microbiota is involved in the development of T2DM and IR through different mechanisms including imidazole propionate, trimethylamine oxide, short-chain fatty acids, and BAs (91, 92). The gut microbiota, along with its metabolites and nutrients, is continuously transported to the liver through the portal blood circulation. Given that the liver is central to glucose and lipid metabolism, T2DM inevitably results in significant alterations in hepatic metabolites and metabolic pathways (93). Of these mechanisms, the BAs pathway is closely linked to liver function and plays a crucial role in regulating glucose and lipid metabolism balance (44). The gut microbiota plays a pivotal role in the biotransformation and reabsorption of BAs, exerting profound effects on host glucose, lipid, and energy metabolism through co-metabolism with BAs (2, 7). This intricate interplay necessitates a deeper understanding of how the gut microbiota intersects with BA metabolism in the context of T2DM and IR.

### 4.1 The role of gut microbiota in BAs

The biotransformation function of gut microbiota plays a crucial role in determining the concentration of each component within the total pool of BAs. Variations in gut microbiota community structure can lead to significant alterations in the composition of the BAs pool. The gut microbiota extensively diversify and enhance the biological functions of these compounds through various transformations. Specifically, microorganisms can modify human BAs in four distinct manners, including glycine or taurine uncoupling, dehydroxylation, dehydrogenation, and emisomerization of the cholesterol core (51). The gut microbiota first fine-tunes BAs metabolism by regulating bile saline lyase (BSH) activity (94, 95). BSH enzyme, as a key enzyme of gut microbiota to modify BAs, is widely distributed in different species of gut microbiota, especially in the genus *Bacteroides (* 96). Animal experiments have elucidated the various roles of *Bacteroides* species in BAs metabolism with contrasting effects on metabolic health. On one hand, it has been observed that patients with a higher proportion of *bacteroidetes* in their gut microbiota experience greater improvements in lipid levels, body weight, and IR following acarbose treatment (97). *Bacteroides* species have been shown to ameliorate obesity, IR, and lipid metabolism disorders in both high fat diet-induced obese mice and ob/ob mice through the modulation of LCA and UDCA levels (98). This highlights their potential as beneficial modulators of metabolic dysfunction. On the other hand, a distinct enrichment of the *Bacteroidia* class has been linked to the severity of T2DM and disruptions in glucose homeostasis markers (99). Specifically, high-fat diet-fed mice colonized with *Bacteroides fragilis* show exacerbated glucose intolerance and abolished metformin efficacy in improving glucose tolerance, accompanied by a decrease in intestinal *Bacteroides fragilis* and an increase in glycoursodeoxycholic acid levels (73). Moreover, in a hypothermia animal model study, the administration of aqueous extracts of Aconite was observed to modulate the gut microbiota and BAs metabolism, resulting in an elevation of body temperature in mice. The findings demonstrated that Aconite extracts facilitated the browning process of white adipose tissue and enhanced BAT expression, thereby augmenting glucose energy metabolism. Importantly, these effects were attenuated by depletion of gut microbiota and reduction in BAs levels induced by antibiotic treatment, indicating that Aconite extracts ameliorated T2DM through precise modulation of gut microbiota (100). The gut microbiota’s biotransformation of BAs is vital in metabolic health, with variations in its community structure significantly altering BAs composition and impacting metabolic functions.

### 4.2 The impact of BAs on the intestinal tract

In the intricate gut ecosystem, BAs play an indispensable role as crucial defenders of the chemical barrier. Conjugated BAs, such as taurocholate, possess unique high solubility properties that not only maintain optimal acidic pH balance in the intestinal lumen but also significantly enhance the activity of intestinal digestive enzymes to ensure efficient nutrient absorption (101–103). This physiological process not only establishes a robust chemical defense barrier for intestinal health, but also lays the groundwork for normal intestinal function. However, this delicate balance is significantly disrupted when the body encounters a state of hyperglycemia. Hyperglycemia facilitates bacterial migration and interferes with the normal secretion and metabolism of BAs, resulting in fluctuations in BAs content within the intestinal lumen. BAs directly target lipopolysaccharide and facilitate the decomposition of endotoxin into non-toxic subunits or the formation of micropolymers (104, 105). They modulate the expression of tight junction proteins, thereby bolstering the integrity of the intestinal mucosal barrier and effectively thwarting the invasion of harmful substances (106). BAs contribute to maintaining the balance of the gut microbiota by inhibiting the excessive proliferation of harmful bacteria. This mechanism involves precise regulation of bacterial proliferation, morphology, membrane permeability, and energy metabolism (107, 108). It is important to emphasize that various types of BAs manifest unique biological effects in maintaining intestinal homeostasis (109–111). For instance, CDCA demonstrates remarkable efficacy in repairing inflammation-induced damage to the intestinal epithelium, thereby reversing the observed increase in transepithelial resistance and decrease in expression of tight junction proteins induced by lipopolysaccharide (112). DCA, LCA, and their derivatives effectively prevent heightened intestinal permeability (113). Moreover, UDCA facilitates migration and repair of damaged intestinal epithelial cells while safeguarding the intestine against further harm caused by detrimental substances like lipopolysaccharides (114). In addition, the TCM prescription Tong-Xie-Yao-Fang in the treatment of irritable bowel syndrome has also demonstrated the unique curative effect. The mechanism of Tong-Xie-Yao-Fang involves the regulation of BAs metabolism in the gut, specifically targeting the synthesis and excretion levels of BAs in irritable bowel syndrome rats. This modulation reduces activation of the colonic BAs membrane receptor Takeda G protein-coupled receptor 5 (TGR5) sensing and its mediated Calcitonin gene-related peptide-positive neuronal response were attenuated, thereby contributing to alleviating symptoms associated with irritable bowel syndrome (115). Pien Tze Huang, a Class 1 nationally protected TCM, has the capacity to activate TGR5 via BAs, thereby exerting its anti-inflammatory effects, enhancing intestinal barrier function, and inhibiting pro-inflammatory pathways (116, 117). TCM demonstrates the therapeutic potential of modulating BAs metabolism in maintaining gut health.

## 5 The gut microbiota-BAs axis affects glucose and lipid metabolism and insulin sensitivity

Diet, genetics, and the environment intricately influence gut microbiota and BAs metabolism, significantly impacting metabolic health. Dietary interventions, particularly focusing on fiber supplementation and specific dietary patterns, show promise in modulating these processes. Dietary factors, especially the Western diet, disrupt the BA profile and contribute to metabolic disorders like T2DM. Conversely, specific dietary interventions can positively influence gut microbiota, BAs metabolism, and metabolic health. The Western diet reduces cecal secondary BAs in mice, but oligofructose supplementation prevents this reduction and elevates 6α-hydroxylated BAs by maintaining key bacteria (118). Oligofructose, a well-recognized soluble fiber, functions as a prebiotic, metabolized by gut bacteria to produce short-chain fatty acids, exerting beneficial effects on glucose lowering, HbA1c, and HOMA-IR in T2DM patients (119). The low-carbohydrate ketogenic diet is commonly used for weight loss. In both an observational study with healthy participants (n = 416) and an intervention study with overweight or obese individuals following this diet, it was found that the diet reduced the abundance of bacteria encoding BSH in the gut microbiota, such as *Lactobacillus murinus*. This led to increased levels of taurodeoxycholic acid and tauroursodeoxycholic acid in circulation, resulting in reductions in body weight and fasting blood glucose levels (120). In addition, a study reveals that a high-fat diet elevates specific gut bacteria in mice, thereby altering the bile acid pool and triggering inflammation. Non-classic amino acid conjugation of the bile acid cholic acid affects intestinal stem cell replenishment, a process mediated by *Ileibacterium valens* and *Ruminococcus gnavus*, which synthesize this specific conjugate (121). These highlights the potential of dietary interventions in modulating BAs metabolism and improving metabolic health. High intake of fatty animal foods increases BAs secretion and is associated with elevated levels of fecal secondary BAs (122, 123). This change may also enhance glucose-6-phosphate dehydrogenase activity which suggests promotion of the pentose phosphate pathway resulting in reduced equivalents (124). The examples provided demonstrate the intricate relationship between diet, gut microbiota, and metabolic processes, highlighting the potential of targeted dietary strategies in mitigating metabolic disorders.

Understanding the genetic and environmental influences on the gut microbiota and BAs metabolism is crucial for developing effective strategies to maintain or improve metabolic health. Building on the observed correlation between gut microbiota structural variation of genome and BAs levels, recent research by Meng et al. further elucidates how antibiotic-induced gut dysbiosis impacts host transcriptome and m(6)A epitranscriptome through BAs metabolism (125, 126). In addition to intrinsic genetic factors, extrinsic environmental factors also exert profound effects on the gut-liver axis, particularly through their interaction with the gut microbiome. Environmental toxins have been linked to gut microbiome dysbiosis and IR, with microbiome-derived secondary BAs potentially acting as mediators between environmental toxins and obesity or IR (127). Lifestyle-induced weight loss does improve glycemic control and IR but does not affect BAs concentrations (128). However, during weight regain, the gut microbiota and BAs play pivotal roles, and supplementation with Parabacteroides distasonis and non-12α-hydroxylated BAs can effectively mitigate weight rebound (129). Thus, understanding these interactions is essential for developing strategies to improve metabolic well-being.

Metabolomics is a potent tool for the precise analysis of metabolites at the molecular level, uncovering disease-induced alterations in metabolites. Monitoring the composition of the human gut microbiota or gut metabolites may provide valuable insights into the diagnosis and treatment of T2DM (130). The BAs pool consists of a variety of BAs subtypes, each with its own unique affinity for different BAs receptors. As a result, BAs play a crucial role in regulating host health by interacting with receptors of varying affinities (131). This interaction not only facilitates the reduction of bile salt load, but also significantly enhances insulin sensitivity, thereby regulating glucose metabolism and controlling energy expenditure (132). The serum BAs concentration is significantly higher in patients with T2DM compared to non-diabetic individuals, and it shows a positive correlation with IR (133). Alessandro et al. analyzed plasma samples from 224 patients with T2DM and 102 nondiabetic individuals with metabolic syndrome, assessing a total of 14 plasma BAs species. This study demonstrates significant disparities in plasma BA profiles between individuals with and without T2DM (134). Chen et al. examined serum samples from 30 individuals and fecal matter from 15 patients to investigate changes in BAs and gut microbiota within the biological milieu of T2DM patients. Their findings reveal a dynamic interaction between BAs and gut microbial populations, highlighting notable elevations in DCA, LCA, and glycodeoxycholic acid levels among the T2DM group, while glycoursodeoxycholic acid levels were significantly reduced (135). Significant changes were reported in the levels of BAs metabolites in the liver of T2DM-IR mice (136). T2DM with IR often presents with inflammation and liver damage (137). Low-level inflammation is thought to be a key factor in the pathogenesis of IR, T2DM, and beta cell impairment (138). Research has demonstrated significant differences in the serum BAs profiles between healthy individuals and patients with intestinal inflammation (109). Taurocholate Deoxycholate has demonstrated efficacy in mitigating inflammation and enhancing insulin sensitivity (139, 140). Subsequent to continuous administration of glycoursodeoxycholic acid, a notable decrease in blood insulin levels and HOMA-IR was observed in db/db mice (34). It is important to note that moderate BAs supplementation can significantly reduce inflammation in the body and effectively improve insulin sensitivity (141, 142).

**Figure 2:**
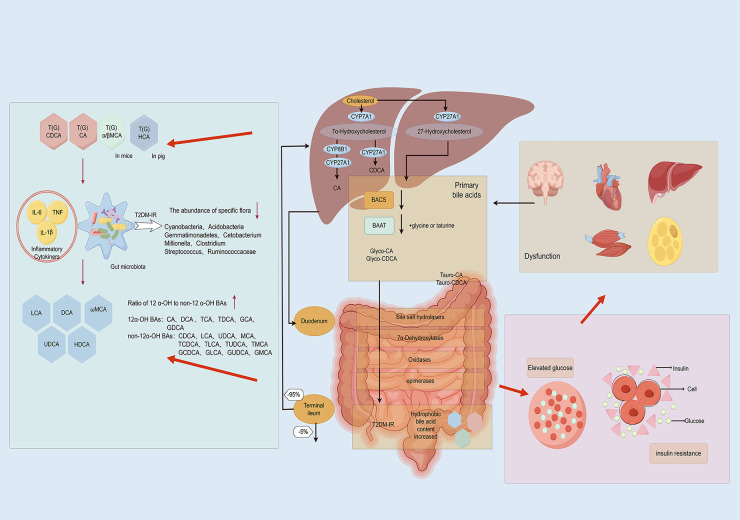
Relevance of gut microbiota-mediated BAs metabolism in the development of IR in individuals with type 2 diabetes mellitus(By Figdraw). Patients with type 2 diabetes mellitus often exhibit intestinal inflammation, resulting in increased intestinal permeability that allows bacterial lipopolysaccharides and BAs to directly enter the liver through the portal vein. Within the intestinal microecosystem, a series of biochemical processes such as uncoupling, dehydrogenation, dehydroxylation, and differential isomerization convert BAs into secondary BAs. As the primary organ responsible for BAs synthesis and metabolism, the liver may alter its synthesis and secretion under inflammatory stimulation, thereby influencing the metabolic balance of BAs. Disordered BAs metabolism can lead to elevated glucose levels and IR, further exacerbating pathological progression.

Bear bile has been used in TCM for thousands of years (143). UDCA, the active ingredient in bear bile, was found to have metabolic, anti-inflammatory, and antioxidant effects in patients with T2DM in a prospective, double-blind, placebo-controlled clinical study (144, 145). Scutellaria baicalensis, a TCM, has been found to modulate the profile of BAs in the intestine through its total flavonoid components, which not only shapes the composition of the intestinal microbiome by promoting the growth of beneficial bacteria and inhibiting harmful bacterial proliferation, thereby optimizing BAs metabolism, but also enhances intestinal barrier function and reduces inflammation caused by BAs accumulation, potentially alleviating IR in T2DM patients (146). Furthermore, Forsythia, a traditional Chinese medicinal herb renowned for its therapeutic properties including clearing heat, detoxification, reducing inflammation, and dispelling wind-heat, has been subject to recent research revealing that its active compound, Phillyrin, shows promise in improving IR (147). Additionally, the study indicates that, when compared to green Forsythia, mature Forsythia may exert more pronounced effects on detoxification and BAs metabolism. This intriguing discovery implies that Forsythia, through its influence on the gut microbiota, could indirectly regulate BAs metabolism, which would ultimately lead to a positive impact on IR in patients with T2DM (148). In a retrospective cohort study involving 147 patients, the Jiang-Tang-San-Huang pill demonstrated significant efficacy in lowering blood glucose levels and reducing IR, thereby enhancing pancreatic islet cell function (149). The therapeutic effects of Jiang-Tang-San-Huang pill are mediated through the correction of gut microbiota imbalances, particularly by enriching bacteria with BSH activity, including *Bacteroides*, *Lactobacillus*, and *Bifidobacterium*. This enrichment facilitates the accumulation of unconjugated BAs in the ileum (150). Ultimately, the Jiang-Tang-San-Huang pill alleviates T2DM-IR by modulating the complex interplay between gut microbiota and BAs metabolism. The research conducted on Jingangteng capsule in diabetic rat models demonstrated that it possesses a significant ability to regulate BAs metabolites linked to BAs receptors and reverse the diminished expression of BAs receptors in the liver. This regulatory mechanism not only downregulates the expression of lipogenesis-related genes but also inhibits the activation of inflammation-related genes, actions which collectively contribute to reducing the inflammatory burden on the liver and intestines, thereby improving IR in patients with T2DM (151). Ji-Ni-De-Xie, a traditional herbal formulation utilized in Tibetan medicine for treating T2DM, has exhibited notable effects in ameliorating IR through modulation of BAs metabolism. The study indicates that Ji-Ni-De-Xie can adjust the composition and concentration of BAs in the intestines, optimize their distribution and function, reduce potential damage to the intestinal mucosa, and enhance nutrient absorption and utilization (152). It’s worth noting that electroacupuncture can improve the disorder of glucose and lipid metabolism in db/db mice, increase the abundance of the *Firmicutes* and *Actinobacteria*, and elevate the content of fecal BAs pool, particularly CA and UDCA (132). These comprehensive mechanism effectively alleviates IR, hyperglycemia, hyperlipidemia, and inflammatory responses, offering new scientific evidence for integrating traditional Chinese medicine into T2DM treatment. Relevance of gut microbiota-mediated BAs metabolism in the development of IR in individuals with T2DM in **Figure 2**.

## 6 T2DM-IR and BAs-related signaling pathways

BAs are a unique class of signaling molecules that can activate a variety of nuclear and membrane receptors (153). Over the past three decades, the scientific community has conducted extensive and comprehensive research into the regulatory functions of BAs receptors in maintaining health and driving disease progression (9, 55, 154). In recent years, studies have revealed that BAs exhibit remarkable efficacy in lowering blood glucose levels through multiple signaling pathways (140, 155, 156). Specifically, it is through the activation of key receptors, such as FXR and TGR5, that BAs profoundly influence numerous physiological processes, which in turn encompass the stimulation of GLP-1 secretion in the intestine, the inhibition of hepatic gluconeogenesis, an increase in energy expenditure, and the modulation of inflammatory responses and gut microbiota composition (157, 158). All of these effects play significant roles in the pathogenesis of IR and T2DM (159, 160).

### 6.1 Membrane receptor TGR5

TGR5, a crucial membrane receptor for BAs, exhibits high expression in the gallbladder, liver, intestinal tract, and adipose tissues. Functioning as a BAs receptor, TGR5 can respond to all known BAs irrespective of their binding state. Studies have shown that secondary BAs exhibit markedly higher affinity for TGR5 than primary BAs, with LCA emerging as the most potent natural agonist for TGR5 (159, 161). Activation of this receptor demonstrates anti-inflammatory properties and aids in preventing chronic inflammation (162). Inflammatory factors have the potential to disrupt insulin signaling pathways through endocrine or paracrine mechanisms, thereby impacting its transduction activity and ultimately leading to T2DM-IR (163).

In the liver, TGR5 is predominantly localized in sinusoidal endothelial cells, Kupffer cells, and cholangiocytes (164). Upon bacterial infiltration into the liver via bloodstream, their surface antigens can trigger macrophage activation leading to the production of proinflammatory cytokines including interferon-γ, interleukin-1β, interleukin-6, and tumor necrosis factor-α. These cytokines promote polarization of M0 macrophages towards a proinflammatory M1 phenotype (165). However, activation of TGR5 has been shown to inhibit nuclear factor kappa-B (NF-κB) signaling pathway thereby attenuating inflammation in Kupffer cells and facilitating transition from an M1 proinflammatory phenotype to an anti-inflammatory M2 phenotype (166, 167). Notably, NF-κB signaling pathway represents a pivotal regulatory axis governing immune response gene expression closely associated with IR in T2DM (168). Qi et al. revealed that taurochenodeoxycholic acid (TCDCA) exerts critical functions in apoptosis and immune responses via the TGR5 receptor, ultimately regulating NF-κB activity (169). In the intestine, cyclic adenosine monophosphate (cAMP) acts as a secondary messenger and is positively regulated by TGR5, leading to rapid and dose-dependent elevation of cAMP levels. Activation of TGR5 leads to an increase in cAMP levels, subsequently stimulating protein kinase A (PKA) and inducing NOD-like receptor family pyrin domain-containing 3 (NLRP3) ubiquitination, which ultimately inhibits NLRP3 inflammasome activation. The NLRP3 inflammasome is pivotal in chronic inflammation and is intimately linked to insulin signaling, glucose tolerance, and IR (170). This mechanism was validated in a mouse model, where BAs were found to alleviate various inflammatory diseases, including high-fat diet-induced T2DM (154). Notably, a modified Gegen Qinlian decoction has been reported to effectively regulate gut microbiota, improve BAs metabolism, and activate the TGR5/cAMP/PKA signaling pathway, thereby exerting therapeutic benefits in mouse models of T2DM (171). Moreover, the research expounded upon the robust connection between TGR5 and GLP-1. Intriguingly, approximately 75% of GLP-1-generating intestinal endocrine cells within isolated human intestinal cells demonstrated expression of TGR5 (156). The activation of TGR5 instigated the discharge of GLP-1 from intestinal endocrine cells, subsequently activating the GLP-1R/PKA signaling pathway and markedly reducing the HOMA-IR index (172, 173). As a crucial intermediary molecule, the influence of GLP-1 transcended the intestine and encompassed various body regions, effectively mitigating peripheral IR (174). In adipocytes, elevated levels of TCA and DCA could activate TGR5. Upon activation, TGR5 stimulated UCP1 expression, thereby fostering increased heat production in white fat and contributing to the improvement of IR (41, 175, 176). The research further substantiated an inverse correlation between UCP1mRNA content and IR, specifically indicating that a higher content corresponded to a lower degree of IR (177). AMPK, a kinase activated by PKA, has been implicated in the onset of metabolic syndrome, including obesity and hyperglycemia (178). Vaccarin, a flavonoid derived from vaccaria seeds and traditionally employed in Chinese medicine to promote blood circulation, has recently been demonstrated to exert anti-diabetic effects (179). It has been demonstrated that vaccarin stimulates the growth of *Bacteroides uniformis* both *in vitro* and *in vivo*, resulting in the production of CA and CDCA that act through the TGR5/AMPK signaling pathway to inhibit hepatic gluconeogenesis and lipolysis, thereby ameliorating diabetes and its potential complications (180). The findings of this study not only enhance the understanding of the intricate relationship between TGR5 and IR but also provide new insights into potential therapeutic interventions for treating IR-related disorders, such as T2DM.

### 6.2 Nuclear receptor FXR

As a crucial nuclear receptor, FXR is predominantly expressed in the intestinal and hepatic tissues, playing an indispensable role in maintaining homeostasis of BAs, lipids, glucose, and energy (181). Natural agonists of FXR such as CA and CDCA have been demonstrated to activate its function (182). In the db/db mouse model of T2DM characterized by IR, treatment with BAs or specific agonists for FXR significantly improved insulin sensitivity and effectively reduced blood glucose levels thereby ameliorating diabetic symptoms (183). The FXR receptor plays a crucial role in the regulation of diverse metabolic pathways, encompassing BAS synthesis, excretion, reverse cholesterol transport, gluconeogenesis, and glycogenolysis (95, 184, 185).

Increased expression or transcriptional activity of FXR in the liver led to a significant reduction in blood glucose levels and improvement in IR, whereas surprisingly, absence of FXR expression or inhibition of its transcriptional activity in the intestine also resulted in decreased blood glucose levels and improved IR (186, 187). This discovery unveils the intricate regulatory mechanism of FXR across different tissues. The FXR/SHP and FXR/FGF15/19/FGFR4 pathways have been identified as the principal negative regulatory pathways in BAs synthesis (133). In intestinal tissues, FXR stimulates the synthesis of fibroblast growth factor 15/19(FGF15/19)and effectively inhibits the expression of CYP7A1 and CYP8B1, thereby regulating BAs synthesis. As an antagonist of FXR, UDCA significantly inhibits the transcription activity of FXR induced by CDCA, highlighting the crucial role of FXR in metabolic regulation (73). By suppressing the signaling pathway of FXR, the release of FGF15/19 is diminished, thereby impeding its binding to fibroblast growth factor receptor 4 (FGFR4) on hepatocytes via the portal vein. This attenuation weakens the inhibitory effect on CYP7A1 and CYP8B1, resulting in an elevation in hepatic BAs synthesis and cholesterol utilization. Consequently, this phenomenon contributes to ameliorating obesity and IR (158). Berberidis Cortex, a traditional Tibetan herbal remedy, ameliorates BAs dysregulation in T2DM rats, notably by elevating levels of TCDCA and CA, subsequently activating the FXR/FGF15 signaling pathway and inhibiting hepatic gluconeogenesis (188).

Another significant aspect is increased hepatic BAs levels and FXR activation induce the expression of small heterodimer partner (SHP) gene which inhibits transcriptional activity of CYP7A1, a key enzyme involved in BAs production, thereby enhancing fatty acid oxidation via peroxisome proliferator-activated receptor alpha (PPARα) activation (189–191). Activation of PPARα leads to reduced blood lipid levels and plays a crucial role in glucose homeostasis as well as IR management (192). In a rat model of T2DM, Roux-en-Y gastric bypass surgery elevated serum BAs levels, which subsequently activated the FXR-related pathway, leading to upregulation of FXR, SHP, and PPARα expression that inhibited adipogenesis and modulated hepatic gluconeogenesis (193). Recent investigations further elucidate the intricate interplay of these pathways in modulating glucose metabolism and insulin sensitivity, exemplified by a study demonstrating that a degraded sweet corn cob polysaccharide ameliorates T2DM through the modulation of BAs synthesis and hepatic lipid metabolism, a mechanism that involves the activation of the hepatic FXR/SHP pathway and the engagement of the FXR/FGF15/FGFR4 signaling axis within the gut-liver axis (194). Furthermore, SHP, a downstream target gene regulated by FXR, has been shown to inhibit NLRP3 inflammasome formation along with downstream inflammatory factors resulting in decreased IR (170, 195). A novel fasting therapy has been found to ameliorate high-fat diet- and STZ-induced IR in rats by suppressing NLRP3 inflammasome-mediated inflammatory response (196). In addition, the role of GLP-1 warrants attention. Oral administration of UDCA not only stimulates GLP-1 secretion but also effectively improves symptoms related to glucose intolerance and IR by inhibiting the FXR signaling pathway (197). Similarly, a single-center cohort study revealed that intestinal Clostridium inhibits the conversion of CDCA to UDCA, leading to a reduction in GLP-1 secretion and subsequently resulting in abnormal glucose metabolism and IR (197). In intestinal L-cells, FXR disrupts the interaction between glucose-responsive transcription factor carbohydrate-responsive element-binding protein (ChREBP) and its target sequences, thereby inhibiting glycolysis and resulting in a reduction in proglucagon expression and, consequently, a decrease in GLP-1 secretion (198).

**Figure 3:**
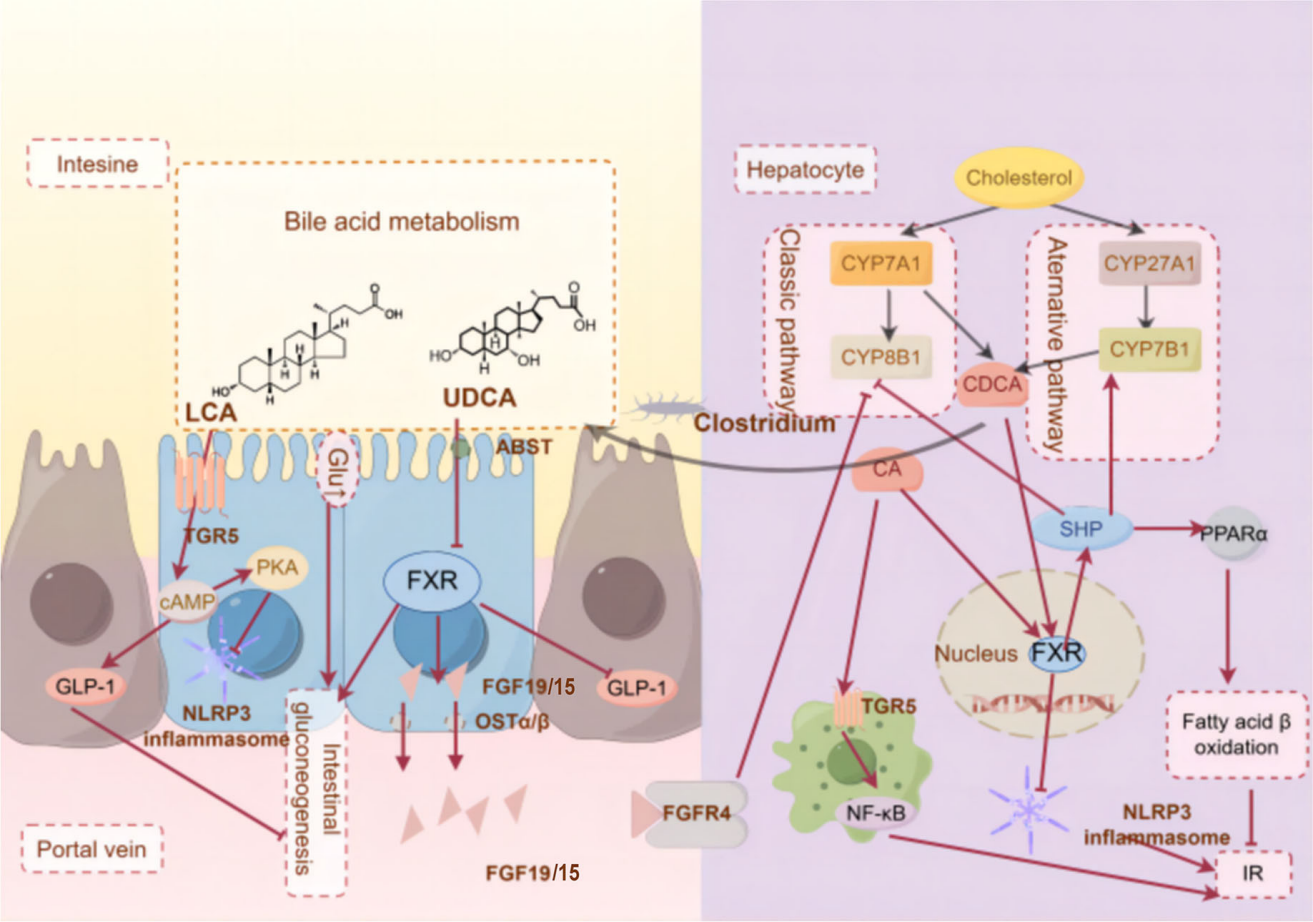
The molecular mechanism of BAs receptors ameliorate T2DM-IR.(By Figdraw)FXR and TGR5 are key BAs receptors that are highly expressed in tissues such as the liver and intestine, responding to bile acid pool composition and inhibiting inflammation, helping to prevent insulin resistance. These metabolites can act on the gut and liver through different signaling pathways, thus triggering different physiological and biochemical reactions.

Nevertheless, studies have demonstrated an intricate interplay between the bile acid receptors FXR and TGR5. Curcumin and Notoginsenoside Ft1, acting as a natural TGR5 agonist and an intestinal FXR inhibitor, exerts a dual effect that alleviates metabolic disorders by activating TGR5 while simultaneously suppressing FXR (199, 200). Additionally, the traditional medicinal formula Jiang-Tang-San-Huang pill upregulates the intestinal FXR/FGF15 and TGR5/GLP-1 signaling pathways (150). In another context, Mulberry leaf polysaccharides have been shown to significantly reduce fasting blood glucose and lipid levels, improve glucose and lipid metabolism, and mitigate IR, and these effects are mediated through the modulation of BAs metabolism, as evidenced by increased ileal expression of TGR5 and suppressed hepatic and ileal expression of FXR mRNA in T2DM rats (201). Collectively, these findings underscore the therapeutic potential of targeting the FXR/TGR5 axis through natural compounds and traditional medicines for the management of metabolic diseases. FXR and TGR5 are summarized in **Figure 3**.

### 6.3 Other receptors

The liver, as the central organ for glucose metabolism, plays a crucial role in maintaining energy homeostasis within the body. In recent years, pregnane X receptor (PXR), a member of the nuclear receptor superfamily, has been identified to be involved in regulating hepatic sugar metabolism and is closely associated with the development of obesity and IR (202, 203). PXR exhibits abundant expression in both the liver and intestine, where it governs the expression of downstream target genes (204). The activation of PXR receptors was observed to induce an up-regulation of the lipid receptor Peroxisome proliferators-activated receptors γ in the liver, thereby promoting hepatocyte steatosis (205). This effect further exacerbates hepatic burden and may lead to hepatic IR. Furthermore, PXR knockout mice fed a high-fat diet exhibit inhibited weight gain, hepatic steatosis, IR, and gluconeogenesis (206). The Vitamin D Receptor (VDR) receptor, which is highly expressed in islet β cells, plays a significant role in reducing islet inflammation, protecting β cell function, and preventing T2DM (207). Moreover, activation of the VDR receptor in liver macrophages not only alleviates inflammation and liver steatosis but also improves insulin sensitivity and effectively addresses IR (208).

## 7 Limitation

BAs, due to their intricate associations with lipid and glucose metabolism, have emerged as a focal point in the realm of metabolic disease research. Extensive studies have eloquently elucidated the pivotal role of BAs in glycolipid metabolism, and a thorough dissection of their physiological and pathological alterations is paramount to unraveling the underlying mechanisms of IR and T2DM (130, 158, 209). Nonetheless, the exploration of the impact of BAs on glycemic responses in both healthy and diseased states in humans remains fraught with numerous challenges and limitations especially at the level of TCM research. BAs are not a singular molecular entity but rather a complex system comprised of diverse metabolites, each possessing distinct biological activities. The mechanisms of action of these metabolites in glucose metabolism are varied and, in part, remain uncharted. Secondly, species differences pose another significant barrier. Fundamental disparities in BAs biology exist between mice and humans, complicating the direct translation of findings from mouse models to human applications (63). This cross-species divergence amplifies the difficulty in converting experimental results into clinical practices. Furthermore, the intricate individual variability of the human gut microbiome profoundly influences the diversity of gut microbial community structures, subsequently shaping unique BAs metabolic profiles (37). This variability serves as a significance for the progression of diseases and the variability in responses to BAs regulatory therapies. This phenomenon closely aligns with the concept of “constitution” in TCM, reflecting deep-seated differences in the physiology and pathology of individuals (210). In the face of challenges posed by individual variability in the practical application of BAs-related treatment strategies, TCM, with its unique approach to constitutional identification and treatment philosophy, offers new perspectives and possibilities for precision medicine. While TCM demonstrates potential in modulating gut microbiota and influencing BAs metabolism, evidence for the effective translation of animal model findings to human clinical trials is currently lacking. This underscores the need for enhanced interdisciplinary collaboration and optimized research designs in future studies to better evaluate the efficacy and safety of TCM in treating T2DM and IR through the gut microbiota-BAs axis. In summary, despite some progress, research on BAs as a key target for treating T2DM faces numerous limitations. Future studies must adopt more precise and personalized strategies to address these challenges, driving further advancements in this field.

## 8 Conclusions

The regulatory role of BAs in the gut-liver axis is of central importance for alleviating IR in T2DM. Current research has delved into the roles of the bile acid nuclear receptor FXR and the membrane receptor TGR5 in T2DM, with some studies in TCM also focusing on this area, emphasizing the potential of TCM in regulating BAs metabolism. Despite these advancements, the specific alterations in BAs levels, abnormalities in their composition, and their association with the pathogenic mechanisms in T2DM remain to be thoroughly explored. TCM boasts a long history and remarkable efficacy in the treatment of T2DM, highlighting holistic regulation and multi-target synergistic effects. Regrettably, in the realm of TCM research targeting T2DM through BAs regulation, there is a notable dearth of adequate modern scientific validation. Notably, the holistic and dynamic nature emphasized by metabolomics research on BAs and gut microbiota aligns well with the “concept of holism” in TCM, offering the potential for scientific explanations of multi-target and multi-pathway mechanisms of action. By taking the BAs metabolic pathway as an entry point and dissecting the complex mechanisms of TCM in T2DM treatment at a microscopic level, we may gain new research perspectives and methodologies, fostering deeper integration of Chinese and Western medicine. There is an urgent need to identify more effective strategies that precisely regulate and utilize BAs metabolism to improve IR in T2DM patients, thereby paving new avenues for the prevention and treatment of this global disease and significantly enhancing health status and quality of life.
